# Antibiotic Exposure of Critically Ill Children at a Tertiary Care Paediatric Intensive Care Unit in Switzerland

**DOI:** 10.3390/children11060731

**Published:** 2024-06-15

**Authors:** Anica Fenner, Melanie Huber, Verena Gotta, Vera Jaeggi, Luregn J. Schlapbach, Philipp Baumann

**Affiliations:** 1 Department of Intensive Care and Neonatology, University Children’s Hospital Zurich, University of Zurich, 8032 Zurich, Switzerland; anica.fenner@uzh.ch (A.F.); melanie.huber@kispi.uzh.ch (M.H.); luregn.schlapbach@kispi.uzh.ch (L.J.S.); 2 Children’s Research Centre, University Children’s Hospital Zurich, University of Zurich, 8032 Zurich, Switzerland; 3 Department of Paediatric Pharmacology and Pharmacometrics/Paediatric Clinical Pharmacy, University of Basel Children’s Hospital, 4056 Basel, Switzerland; verena.gotta@ukbb.ch; 4 Department of Data Intelligence, University Children’s Hospital Zurich, University of Zurich, 8032 Zurich, Switzerland; vera.jaeggi@kispi.uzh.ch

**Keywords:** neonate, infant, child, children, antibiotics, antimicrobials, critically ill patients, intensive care, infection

## Abstract

Antibiotic overtreatment fosters multidrug-resistance that threatens healthcare systems worldwide as it increases patient morbidity and mortality. Contemporary data on antibiotic usage on tertiary care paediatric intensive care units for in- and external benchmarking are scarce. This was a single-centre retrospective quality control study including all patients with antibiotic treatment during their hospitalization at a paediatric intensive care unit in the time period 2019–2021. Antibiotic treatment was calculated as days of therapy (DOT) per 100 patient days (DOT/100pd). Further, the variables PIM II score, length of stay in intensive care (LOS), gender, age, treatment year, reason for intensive care unit admission, and death were assessed. Two thousand and forty-one cases with a median age of 10 months [IQR 0–64] were included; 53.4% were male, and 4.5% of the included patients died. Median LOS was 2.73 days [0.07–5.90], and PIM II score was 1.98% [0.02–4.86]. Overall, the antibiotic exposure of critically ill children and adolescents was 59.8 DOT/100pd. During the study period, the antibiotic usage continuously increased (2019: 55.2 DOT/100pd; 2020: 59.8 DOT/100pd (+8.2%); 2021: 64.5 DOT/100pd (+8.0%)). The highest antibiotic exposure was found in the youngest patients (0–1 month old (72.7 DOT/100pd)), in patients who had a LOS of >2–7 days (65.1 DOT/100pd), those who had a renal diagnosis (98 DOT/100pd), and in case of death (91.5 DOT/100pd). Critically ill paediatric patients were moderately exposed to antibiotics compared to data from the previously published literature. The current underreporting of antimicrobial prescription data in this cohort calls for future studies for better internal and external benchmarking.

## 1. Introduction

The mortality of critically ill paediatric patients from invasive bacterial infections is high [1] and early antibiotic therapy can be lifesaving [2,3]. As a consequence, many patients are generously and often unnecessarily treated with broad-spectrum antibiotics when invasive bacterial infections are suspected [4]. The main driver seems to be the attending physicians’ fear of missing potentially lethal bacterial infections [5]. This phenomenon, known in many medical specialities, varies internationally but is often associated with relevant antibiotic overtreatment [6,7,8]. The consequence is a contribution to the worldwide development of increasing numbers of multi-resistant microbiota that can only be treated with broad-spectrum reserve antibiotics [9,10,11]. Bacterial antimicrobial resistance (AMR) was identified as one of the major public health threats by the WHO, and one recent systematic analysis reported nearly 5 million deaths associated with AMR as well as 1.3 million deaths attributable to AMR in 2019 [12]. This, in turn, leads to an increased treatment of patients with broad-spectrum antibiotics who are not infected with a multidrug-resistant germ [13]. Inadequate treatment with broad-spectrum antibiotics worsens patient outcomes and is associated with higher mortality [12,13,14,15]. The intensive care unit is considered as one of the hospital departments with the highest antibiotic consumption [16,17]. The reasons include the significant disease severity of patients treated in intensive care, perioperative care with the need for antibiotic prophylaxis, the use of invasive vascular catheters, the prescription of sedatives and relaxant drugs that suppress cough and promote pneumonia, and drugs that affect the protective human microbiome [17].

In recent years, the antibiotic exposure of adult intensive care patients has been well investigated in standardised manner and international data are available: up to 70% of patients in intensive care were treated with antibiotics [10,18]. The data availability for critically ill children is not as good as for adults, but published paediatric studies have shown similarly high treatment rates: 60–80% of all children were treated with antibiotics in intensive care units [4,19,20]. However, paediatric studies showed high regional and inter-institutional variability [21,22], and very few and no recent data have been published for Switzerland [23].

The assessment of paediatric antibiotic use differs from the methods widely used in adults. Adult cohorts and their antibiotic exposure are often measured in Defined Daily Doses (DDDs). DDDs are calculated with WHO-defined drug dosages from the antibiotic consumption reported by the hospital pharmacy. This method is not suitable in childhood because DDDs are not weight-based [24]. To investigate the paediatric antibiotic exposure, the «Days of Therapy» (DOT) method is one of the methods of choice [25]. DOTs are usually related to 100 or 1000 patient days of ICU hospitalisation to enable inter-hospital comparability.

The goal of this study was to retrospectively quantify the antibiotic exposure of children and adolescents admitted to the multidisciplinary level III neonatal and paediatric intensive care unit of the Children’s Hospital Zurich from 2019 to 2021.

## 2. Materials and Methods

This study was designed as a single-centre retrospective quality control study. It was conducted in critically ill children admitted to the 25-bed mixed neonatal and paediatric tertiary intensive care unit of the University Children’s Hospital Zurich. The local Swiss ethics committee approved the quality control nature of this study, and no informed consent was necessary for coded data use (Req-2024-00296).

### 2.1. Participants

All patients, irrespective of age and their medical condition, who were admitted to the mixed neonatal and paediatric intensive care unit (NICU/PICU) of the University Children’s Hospital Zurich between 1 January 2019 and 31 December 2021 and who received at least one dose of antibiotic treatment were included. Every admission was counted as one case. Further, the length of stay of all patients admitted to the unit in the respective period of time was summarized to be used as denominator in the calculation DOT/100pd.

### 2.2. Study Design

All patients admitted to the PICU who received antibiotics in the years 2019–2021 were identified through a keyword search of the electronic medical records in the digital patient data management system (PDMS, Metavision^®^, iMDsoft, Tel Aviv, Israel), using the names of the antibiotic drugs (commercial and substance names) that were delivered by the central hospital pharmacy to the PICU in the respective years. The search results yielded the exact antibiotic application times and dates, which were extracted into a Microsoft^®^ Excel^®^ 2019 list (Microsoft^®^, Redmond, DC, USA). The complete list of keywords (commercial and substance names of antibiotics) used to search the electronic records is available in Supplement S1. Further, baseline variables of identified patients were manually extracted from the hospital documentation system (Phoenix^®^, Aveniq AG, Oftringen, Switzerland). Only data stored during clinical routine in the electronic patient data management and documentation systems of the intensive care units of the Children’s Hospital Zurich (Metavision and Phoenix) were used. The data were extracted, coded, and manually entered into RedCap^®^ (https://www.project-redcap.org/ (last accessed on 30 April 2024). Baseline characteristics were calculated in SPSS^®^ (IBM, Armonk, New York, NY, USA), and DOT calculations were performed in Python^®^ (https://www.python.org/).

### 2.3. Variables

The following baseline variables were extracted manually from electronic medical records on an individual case basis: treatment year (2019, 2020, or 2021), age (in months), gender, length of PICU stay (LOS, in days), severity of illness (Paediatric Index of Mortality II (PIM II, in percent)) score, reason for intensive care admission (primary diagnosis, categorized into neurologic, cardiac, respiratory, trauma, postinterventional, miscellaneous, gastrointestinal, and renal), and death during PICU stay. The length of PICU stay (LOS, in days) for all patients (treated with antibiotics and not treated with antibiotics) hospitalised in the PICU in the years 2019–2021 was provided in cumulative form by the medical controlling department of the University Children’s Hospital Zurich. DOTs were defined according to previously published methods [22,26,27] as follows: each day on which at least one dose of a specific antibiotic drug was administered was counted as one day of treatment (=1 DOT). If multiple doses of one antibiotic were administered on one calendar day, this resulted also in 1 DOT. If one or multiple doses of one antibiotic and one or multiple doses of a different antibiotic were administered on the same calendar day, this resulted in 2 DOTs. If antibiotics were already administered on PICU admission day or the patients were still on antibiotic therapy on day of discharge, the day of admission or the day of discharge were counted as half a day (=0.5 DOT). If two different antibiotic drugs were administered on admission or discharge days, each drug resulted in 0.5 DOT. Only systemic (oral or intravenous) applications were used for DOT calculation, and topical therapies were not considered. DOTs were related to 100 PICU patient days by the formula: (DOT/LOS)*100, with LOS referring to all patients who were admitted to the PICU, regardless of antibiotic treatment. If one patient was admitted to the PICU multiple times, these admissions were counted as different cases.

### 2.4. Endpoint Assessment

The primary endpoint was the overall ratio DOT/100pd for the period between 1 January 2019 and 31 December 2021. The secondary endpoint was the relationship of antibiotic exposure to baseline characteristics. This was evaluated via the calculation of DOT/100pd for each treatment year, age groups (0–1 months, >1–12 months, >1–5 years, >5–12 years, and >12 years), gender, LOS (grouped into 0–2, >2–7, and >7 days), diagnosis group, and death during PICU stay. Normal distribution was evaluated via Shapiro–Wilk test. Data are presented as median [interquartile range, IQR] for non-normally distributed continuous variables, or as absolute number (%) for categorical variables. Antibiotic drugs were grouped into classes of antibiotics according to their chemical structures and according to their WHO AWaRe category [28]. As the DOT/100pd calculation resulted in one single absolute numerical value for each group, inter-group comparisons to detect significant differences between groups were not allowed. The results could be compared directly without further statistical tests.

## 3. Results

In the study period between 1 January 2019 and 31 December 2021, 4071 PICU admissions were recorded, 2041 cases (50.1%) received at least one antibiotic drug (see full distribution of antibiotic drugs in Supplement S2). The total cumulative LOS of all 4071 admitted cases was 19,766 days, and 11,817.5 cumulative DOTs were counted for the 2041 cases with antibiotic treatment. The median age was 10 months [IQR 0–64]. Children from the youngest age category (0–1 month) were most represented, with 587 (28.8%) cases. Both genders were practically equally represented, with 952 (46.6%) female and 1098 (53.4%) male cases. Ninety-one (4.5%) patients died during their stay in the intensive care unit. Overall, the median DOT was 2.0 days [IQR 0.5–4.5], the median LOS was 2.73 days [IQR 0.07–5.90], and the median PIM II-score was 1.98% [IQR 0.02–4.86]. One thousand and six cases (49.3%) were admitted to the PICU following an intervention (surgical or non-surgical). All baseline characteristics are displayed in Table 1.

Twenty-two thousand three hundred and forty-eight single antibiotic administrations were recorded during PICU stay. The class of antibiotic which was most commonly administered were penicillins, with 32.8% of all doses, followed by cephalosporins, with 22.0% (Table 1).

Overall, antibiotic exposure was 59.8 DOT/100pd. The antibiotic exposure in relation to age was highest in cases of the age group 0–1 month (72.7 DOT/100pd), followed by the age group 5–12 months (70.1 DOT/100pd). Females and males had almost equal antibiotic exposure. Cases with a LOS of >2–7 days had the highest exposure (65.1 DOT/100pd) of all length of stay groups. Overall, the highest antibiotic exposures were found in cases who died (91.5 DOT/100pd) and cases who had a renal diagnosis (98 DOT/100pd). There was a yearly increase in antibiotic exposure during the study period (2019: 55.2 DOT/100pd; 2020: 59.8 DOT/100pd (+8.2%); 2021: 64.5 DOT/100pd (+8.0%)). The results are shown in Table 2.

## 4. Discussion

Antimicrobial therapy is fundamental in treating bacterial infections in critically ill children and adolescents. Choosing the right antibiotic regime for each infection still poses difficulties for the treating physicians, and clinicians tend to overuse antimicrobials due to concerns about the perceived threat of a potential infection [29]. Standardized surveys of antibiotic exposure allow healthcare facilities to assess and validate their antibiotic usage. However, data on antibiotic exposure of critically ill children are scarce, and patient populations as well as prescription patterns vary significantly throughout the world. The antibiotic exposure of critically ill children and adolescents is underreported and was unknown for our unit.

We found the overall antibiotic exposure to be moderate with 59.8 DOT/100pd in comparison to published data. A study of nine Canadian hospitals reported an overall paediatric (PICU, NICU, and non-ICU wards) antibiotic exposure of 48.1 DOT/100pd in 2017 and 2018. In that study, antibiotic use was found to be 33.3 DOT/100pd for NICU patients, 49.4 DOT/100pd for the general paediatric wards, and 78.4 DOT/100pd for PICU patients [30]. An Italian study, including all paediatric patients hospitalized in four hospitals in 2016, reported 47.2 DOT/100pd [31]. Antibiotic use seems to be higher in low- and middle-income countries, as a study comparing antibiotic consumption in PICUs across three hospitals in Germany (2018) and Brazil (2016) found average consumption rates of 88.8 DOT/100pd and 144.1 DOT/100pd, respectively. Additionally, antibiotic consumption in NICUs was reported to be 38.7 DOT/100pd in Germany and 133.6 DOT/100pd in Brazil [32]. However, a larger study conducted in the USA included 41 PICUs and reported a median antibiotic exposure of 104.3 DOT/100pd in 2010–2014 [22]. In a single-centre study conducted in Saudi Arabia in 2017, antibiotic consumption in PICU patients was found to be 84.9 DOT/100pd [33], while a similar study conducted in South Africa in 2015 revealed a rate of 113.7 DOT/100pd [8]. The mentioned studies showed large-scale differences in prescribing patterns that might arise from differing demographic characteristics, from variations in infectious diseases and resistance patterns, and from the availability of AMS programs: some institutions might have well-developed programs while others seem to lack effective ones [32]. Regional discrepancies in bacterial antimicrobial resistance (AMR) have been revealed in recent studies. Typically, across high-income countries, there is a trend to elevated antibiotic prescription rates, whereas in low- and middle-income countries, antibiotic underprescribing or insufficient dosing predominates. Both situations contribute to the emergence of antimicrobial resistance. Within Europe, one study investigating antimicrobial consumption shed light on notable differences in antibiotic usage patterns between Northern and Southern Europe. It revealed that the total consumption of antibacterials for systemic use tends to be higher in Southern Europe than in Northern Europe [34]. In Switzerland, the Swissnoso survey examined healthcare-associated infections and antimicrobial use in participating Swiss acute care hospitals from 2017 to 2023, excluding 2020. The survey revealed that in the subset of hospitals participating in all surveys, antimicrobial use was significantly higher both in 2022 and 2023 compared to previous years. Cephalosporins emerged as the most commonly used antibiotic class, with co-amoxicillin being the preferred single antimicrobial agent [35]. The latter was in line with our study, in which we found amoxicillin (±clavulanic acid) to be used most frequently.

In this study, we observed a yearly 8% increase in antibiotic exposure from 2019 to 2021. This finding is alarming and underlines the importance of rigorous AMS programs to effectively implement stewardship measures. Moreover, the exhaustive education of all groups of healthcare workers, including medical students, nurses, physicians, and pharmacists, should target effective and rational antimicrobial use to combat antimicrobial overtreatment. AMS programs are highly prioritized by the World Health Organization (WHO) as they are necessary for protecting the efficacy of antimicrobials and reduce adverse events. While many healthcare providers are aware of recommended AMS strategies, the translation into clinical practice remains challenging. Multidisciplinary teams are able to provide the best available theoretical input into treatment decision making and can share the responsibility of restrictive antibiotic treatment guidance [36,37,38,39,40]. Reports in the literature provide information on successful antimicrobial stewardship implementation in paediatric intensive care. Several studies compared antibiotic exposure before and after stewardship implementation measurements. One study conducted in a PICU in Kuwait from 2018 to 2020 reported a monthly antimicrobial use of 48.5 DOT/100pd after implementation, compared to 92.2 DOT/100pd before implementation [41]. Similarly, a study in Germany, which included one PICU, demonstrated a decrease in antibiotic exposure from 150.7 DOT/100pd in 2017 to 100.0 DOT/100pd in 2018 after the implementation of measures to optimize antimicrobial consumption [42]. During the study period, we had institution-specific antibiotic treatment guidelines and pharmacokinetic/pharmacodynamic surveillance capacities as well as 24/7 support by the infectiology department, but it was not until 2022 that regularly scheduled AMS-team visits (four times per week) to the PICU were implemented. Further, feedback and audit on prescribing volume and accuracy are necessary to foster treatment accuracy, i.e., the right antimicrobial agent according to resistance patterns. This is still in development due to the challenges of the demanding data management and labour-intense work-up of comparing prescribed agents with diagnoses and resistance reports.

In this study, we found the age group with the highest antibiotic exposure to be 0–1 month, with a rate of 72.7 DOT/100pd. Interestingly, in contrast to our findings, other studies mentioned earlier with specific NICU data [30,32] reported antibiotic use in the NICU not exceeding that of their PICU data. NICU wards with higher levels of specialization often report a higher use of antimicrobials, reflecting the complexity of cases treated, including higher rates of complications, severity of illness, and elevated infection risk in extremely premature neonates [30]. Further, our cohort included nearly 50% postinterventional or postsurgical patients, who require postoperative antibiotic prophylaxis for 1–5 days, depending on the intervention. This cohort includes neonates who were operated within the first month of life due to cardiac and abdominal birth defects. Accordingly, the proportion of cephalosporine use, one of the most frequently used antimicrobial groups for perioperative prophylaxis, was high with 22%. As this study targeted prescribing volume and overall antibiotic exposure, we did not differentiate between the treatment of proven infections and antibiotic prophylaxis. Two thirds of the antibiotics prescribed in this study belong to the WHO AWaRe category “Access”, with a low risk for resistance development, but one third belongs to the category “Watch”, that includes broad-spectrum antibiotics with an increased risk for resistance development. Very few patients were treated with drugs belonging to the “Reserve” group. While the latter is a positive result, the relevant volume of “Watch” prescriptions calls for caution not to overprescribe, because resistance rates in children still tend to be low in comparison to adults. However, an assessment of treatment accuracy was not within the scope of this study. Furthermore, our study identified the group of patients who died during their PICU stay as having the highest antimicrobial use, with a rate of 91.5 DOT/100pd. This could be attributed to the more complicated cases that led to death, that were possibly treated with multiple antibiotics to avoid death by infection.

## 5. Limitations

This quality control study has several limitations. First, this study is a retrospective analysis that covered the years 2019–2021 including the outbreak of SARS-CoV-2, which could have influenced antibiotic prescribing. It is worth noting that the COVID-19 pandemic disrupted numerous aspects of the healthcare system, including AMS programs. The complex presentation of the disease, particularly in adults, resulted in increased antimicrobial prescribing and the assessment of antimicrobial appropriateness became more challenging. Furthermore, social distancing measures and reduced interaction among healthcare staff contributed to communication issues and may have impacted exchange between healthcare providers and AMS teams [43]. Second, this study was carried out at a single centre. Because of that, we cannot assume our findings to be transferable to other local institutions or to other healthcare systems. Third, we only assessed data over a three-year period, limiting our ability to evaluate larger temporal changes. During the study period, the AMS program at our hospital included antibiotic guidelines and 24/7 access to infectiology specialists, but no in-person visits from AMS teams. Before–after comparisons are planned for the future at the study hospital. Fourth, cumulative LOS data from the hospital medical controlling department were used for DOT/100pd calculations. This prevented the calculation of individual DOT/pd values and resulted in single numerical DOT/100pd values for each group. Statistical inter-group comparisons were not allowed with this data structure. Last, Switzerland is a high-income country, which may limit the generalizability of our findings to a global scale. While AMS programs are generally labour-intense and often led by physicians, microbiologists, and pharmacists, a shortage of specifically trained personnel in low- and middle- income countries might hinder effective implementation [36].

## 6. Conclusions

This quality control study examined the antibiotic exposure of critically ill children and adolescents at the University Children’s Hospital Zurich from 2019 to 2021. The overall exposure was moderate compared to findings from published international reports and increased yearly, potentially under the influence of the COVID-19 pandemic. As reports on antibiotic use in critically ill children and adolescents are still incomplete, further research, possibly as part of local AMS programs, is required to identify areas of improvement in antimicrobial treatment in this age group.

## Figures and Tables

**Table 1:** Baseline characteristics of the 2041 cases included in the analysis.

Patient Characteristics	Value
Age (months)	10.0 [0, 64]
Age stratification	
0–1 Month	587 (28.8)
>1–12 Months	494 (24.2)
>1–5 Years	431 (21.1)
>5–12 Years	342 (16.8)
>12 Years	187 (9.2)
Gender	
Female	952 (46.6)
Male	1089 (53.4)
Treatment year	
2019	709 (34.7)
2020	658 (32.2)
2021	674 (33.0)
DOT (days)	2.0 [0.5, 4.5]
PIM II (%)	1.98 [0.02, 4.86]
LOS (days)	2.73 [0.07, 5.90]
LOS	
0–2 days	902 (44.19)
>2–7 days	650 (31.85)
>7 days	489 (23.96)
Death	
No	1950 (95.5)
Yes	91 (4.5)
Diagnosis ^1^	
Respiratory	388 (19.01)
Cardiac	200 (9.8)
Gastrointestinal	50 (2.45)
Renal	24 (1.18)
Neurologic	131 (6.42)
Trauma	34 (1.67)
Postinterventional	1006 (49.29)
Miscellaneous	208 (10.19)
Class of antibiotic (%) ^2^	
Penicillin	32.80
Cephalosporin	21.94
Carbapenem	14.23
Aminoglycoside	10.53
Glycopeptide	8.06
Sulfonamide/Diaminopyrimidine	5.40
Other	7.04
AWaRe classification (%) ^3^	
Access	63.07
Watch	36.86
Reserve	0.04

Table 1. Data are presented as median [interquartile range] for continuous variables or number (%) for categorical variables. DOT indicates days of therapy; PIM indicates Paediatric Index of Mortality II-score; LOS indicates length of stay. ^1^ Diagnosis refers to the primary diagnosis which was the reason for admission to intensive care. ^2^ A total of 22,348 doses of antibiotics were given. Penicillin, cephalosporin, and carbapenem belong to the beta-lactam antibiotic class. Penicillin includes amoxicillin/± clavulanate, piperacillin/tazobactam, benzylpenicillin, flucloxacillin, and phenoxymethylpenicillin; cephalosporin includes cefixime, cefpodoxime, cefazoline, ceftazidime, ceftriaxone, and cefuroxime; carbapenem includes meropenem only; aminoglycoside includes tobramycin and gentamicin; glycopeptide includes teicoplanin and vancomycin; sulfonamide/diaminopyrimidine includes trimethoprim/sulfamethoxazole only; other includes metronidazole, azithromycin, erythromycin, clarithromycin, linezolid, pyrazinamide, ethambutol, isoniazid, ciprofloxacin, clindamycin, colistin, doxycycline, and rifampicin. ^3^ AWaRe classification of antibiotics for evaluation and monitoring of use (2023, WHO) [28]; tuberculostatic agents are not included (0.03%).

**Table 2:** DOT/100pd overall and per category.

Category	DOT/100pd
Overall	59.8
Age stratification	
0–1 Month	72.7
>1–12 Months	45.0
>1–5 Years	65.0
>5–12 Years	70.1
>12 Years	62.6
Gender	
Female	60.8
Male	59.1
Treatment year	
2019	55.2
2020	59.8
2021	64.5
LOS	
0–2 days	47.0
>2–7 days	65.1
>7 days	60.6
Death	
Yes	91.5
No	56.8
Diagnosis	
Neurologic	41.5
Cardiac	58.4
Respiratory	58.5
Trauma	58.8
Postinterventional	60.1
Miscellaneous	61.9
Gastrointestinal	92.5
Renal	98.0

DOT = days of therapy; pd = patient days; LOS = length of stay.

## Data Availability

All data generated or analysed during this study are included in this article and in Supplemental Material. Further enquiries can be directed to the corresponding author.

## Supplementary Materials

The following supporting information can be downloaded at: https://www.mdpi.com/article/10.3390/children11060731/s1, Supplementary material.
