# Supplementary material for: Antibiotic Exposure of Critically Ill Children at a Tertiary Care Paediatric Intensive Care Unit in Switzerland
Source: Children (Basel). 2024 Jun 15;11(6):731. doi: 10.3390/children11060731 (PMC11201616; doi:10.3390/children11060731)
Supplement: Supplementary file 1 [file children-11-00731-s001.zip › children-3022710-supplementary.pdf]

Supplement S1

Commercial and substance names used for keyword search in to identify patients who received antibiotics in the paediatric intensive care unit of the University Children`s Hospital Zurich in the years 2019-2021.

- Amoxicillin, Clamoxyl (amoxicillin)
- Bactrim (trimethoprim)
- Cefazolin (cefazolin)
- Cefuroxime (cefuroxime)
- Ciproxin (ciprofloxacin)
- Clindamycin phosphate (clindamycin)
- Co-amoxicillin, Co-Amoxi, Co Amoxi, Amoxilline/Acide clavulanique (amoxicillin/clavulanic acid)
- Colistin (colistin)
- Cotrim Ratiopharm, Nopil (sulfamethoxazole/trimethoprim)
- Erythrocin, Infectomycin (erythromycin)
- Floxapen (flucloxacillin)
- Fortam (ceftazidime)
- Gentamicin (gentamicin)
- Isoniazid (isoniazid)
- Infectoopticef, Cefixoral (cefixime)
- Klacid, Klaciped (clarithromycin)
- Linezolid (linezolid)
- Meronem (meropenem)
- Metronidazole (metronidazole)
- Myambotol (ethambutol)
- Obracin (tobramycin)
- Ospen (phenoxymethylpenicillin/penicillin V)
- Penicillin (penicillin/benzylpenicillin)
- Podomexef (cefpodoxime)
- Pyrazinamide (pyrazinamide)
- Rimactan, Rifadine, Rifampicin (rifampicin)
- Rocephin (ceftriaxone)
- Targocid (teicoplanin)
- Tazobac (piperacillin/tazobactam)
- Tobi, Bramitob (tobramycin)
- Vancomycin (vancomycin)
- Vibravenous, Vibramycin (doxycycline)
- Zithromax, azithromycin (azithromycin)

Supplement S2

Distribution of antibiotics used in the paediatric intensive care unit in the years 2019-2021.

Amoxicillin (± clavulanic acid)	31.35%
Azithromycin	0.12%
Benzylpenicillin	0.01%
Cefazolin	9.94%
Cefixime	0.01%
Cefpodoxime	0.44%
Ceftazidime	8.00%
Ceftriaxone	1.87%

Cefuroxime	1.68%
Ciprofloxacin	0.11%
Clarithromycin	0.05%
Clindamycin	0.75%
Colistin	0.01%
Doxycycline	0.01%
Erythromycin	1.12%
Ethambutol	0.01%
Flucloxacillin	1.09%
Gentamicin	9.86%
Isoniazid	0.01%
Linezolid	0.04%
Meropenem	14.23%
Metronidazole	4.59%
Phenoxymethylpenicillin	0.08%
Piperacillin-Tazobactam	0.27%
Pyrazinamide	0.01%
Rifampicin	0.23%
Teicoplanin	7.28%
Tobramycin	0.67%
Trimethoprim/Sulfamethoxazol	5.40%
Vancomycin	0.78%
